# Effects of polyunsaturated fatty acids on the growth of gastric cancer cells *in vitro*

**DOI:** 10.1186/1476-511X-12-71

**Published:** 2013-05-10

**Authors:** Jinfeng Dai, Junhui Shen, Wensheng Pan, Shengrong Shen, Undurti N Das

**Affiliations:** 1 Department of Food Science and Nutrition, School of Biosystems Engineering & Food Science, Zhejiang University, Hangzhou 310058, China; 2 School of Medicine of Tongji University, Shanghai, 200092, China; 3 Department of Gastroenterology, The Second Affiliated Hospital, School of Medicine, Zhejiang University, Hangzhou, 310009, China; 4 UND Life Sciences, 13800 Fairhill Road, #321, Shaker Heights, OH, 44120, USA; 5 School of Biotechnology, Jawaharlal Nehru Technological University, Kakinada, 533 003, India; 6 Bio-Science Research Centre, Gayatri Vidya Parishad College of Engineering, Visakhapatnam, 530 048, India

**Keywords:** Polyunsaturated fatty acids, Gastric cancer cells, Oxidative stress, *de novo* lipid synthesis, Lipid metabolites

## Abstract

Polyunsaturated fatty acids (PUFAs) have tumoricidal action, though the exact mechanism of their action is not clear. The results of the present study showed that of all the fatty acids tested, linoleic acid (LA) and α-linolenic acid (ALA) were the most effective in suppressing the growth of normal gastric cells (GES1) at 180 and 200 μM, while gastric carcinoma cells (MGC and SGC) were inhibited at 200 μM. Arachidonic acid (AA) suppressed the growth of GES1, MGC and SGC cells and lower concentrations (120 and 160 μM) of AA were more effective against gastric carcinoma (MGC and SGC) cells compared to normal gastric cells (GES1). Paradoxically, both eicosapentaenoic (EPA) and docosahexaenoic (DHA) acids though are more unsaturated than AA, were less effective compared with LA, ALA and AA in suppressing the growth of both normal and cancer cells. At the concentration used, methotrexate showed much less growth suppressive action compared to all the fatty acids tested. PUFAs-treated cells showed accumulation of lipid droplets. A close association was noted between apoptosis and lipid peroxides formed compared to the ability of normal and tumor cells to generate ROS (reactive oxygen species) and induce SOD (superoxide dismutase activity) in response to fatty acids tested and methotrexate. Both normal and tumor cells generated lipoxin A_4_ (LXA_4_) in response to supplementation of fatty acids and methotrexate though no significant correlation was noted between their ability to induce apoptosis and LXA_4_ formed. These results suggest that PUFAs induced apoptosis of normal gastric and gastric carcinoma cells could, partly, be attributed to lipid peroxidation process.

## Introduction

Gastric cancer is the fourth most prevalent malignant disease and the second leading cause of cancer death worldwide [1,2]. Despite significant advances, gastric cancer remains a formidable disease to manage.

There is considerable evidence to suggest that essential fatty acids (EFAs): cis-linoleic acid (LA, 18:2, ω-6) and α-linolenic acid (ALA, 18:3, ω-3) and their metabolites exert significant inhibitory action on the growth of tumor cells both in vitro and in vivo [3-16]. It has been documented that tumor cells have decreased activity of Δ^6^ and Δ^5^ desaturases that are essential for the metabolism of LA and ALA to their respective long-chain metabolites [17-19]. The long-chain metabolites of EFAs: arachidonic acid (AA, 20:4 ω-6), eicosapentaenoic acid (EPA, 20:5 ω-3) and docosahexaenoic acid (DHA, 22:6 ω-3) not only give rise to prostaglandins, leukotrienes and thromboxanes but also to anti-inflammatory compounds lipoxins, resolvins, protectins and maresins [20,21].

Previously, we and others showed that gamma-linolenic acid (GLA, 18:3 ω-6), AA, EPA and DHA could be selectively cytotoxic to various tumor cells in vitro and in vivo [3-16]. Many of these studies were performed without taking into consideration the action(s) of these fatty acids on respective normal cells. Hence, in the present study we examined the effect of various long-chain fatty acids on the growth of gastric carcinoma cells and their respective normal gastric cells. We also studied fatty acid profile of cells supplemented with various fatty acids and their influence on the formation of lipid peroxides and free radical generation.

In addition to the generation of various prostaglandins (PGs), thromboxanes (TXs), and leukotrienes (LTs) from PUFAs, especially from DGLA, AA, and EPA that are pro-inflammatory in nature, AA, EPA and DHA also form precursor to anti-inflammatory compounds such as lipoxins (LXs), resolvins and protectins [20,21]. There is reasonable evidence to suggest that cancer could be a low-grade systemic inflammatory condition [22,23] that is supported by the observation that tumor cells produce significant amount of pro-inflammatory eicosanoids [24-26]. But, it is not known whether tumor cells are capable of producing anti-inflammatory compounds such as LXs, resolvins and protectins and, if so, how supplementation of various PUFAs alters their generation and the relationship between the generation of these anti-inflammatory compounds and tumor cell growth. Hence, in the present study, we also measured the amounts of LXA_4_ generated by normal and gastric cancer cells when supplemented with various PUFAs and the results are reported here.

## Materials and methods

### Materials

LA, ALA, AA, EPA, DHA were obtained from Sigma (St. Louis, MO, USA). The human gastric cancer cell line, MGC (undifferentiated), and normal stomach cell line GES1 were kindly provided by Dr. P. Wensheng (Zhejiang University, Hangzhou, China). Human gastric cancer cell line SGC (semi-differentiated) was obtained from Shanghai Institute of Cell Biology, Chinese Academy of Sciences. RPMI medium 1640 was purchased from GIBCO (Grand Island, NY, USA). As a positive control, anticancer drug methotrexate (MTX) was used. MTT (3-(4, 5-dimethylthiazolyl-2)-2, 5-diphenyltetrazolium bromide) was purchased from Sigma corporation. All other chemicals were of extra-pure grade or analytical grade.

### Cell culture

Gastric cancer cells (MGC and SGC) and normal stomach cell line (GES1) were maintained in RPMI-1640, containing 10% fetal bovine serum and 100 U/ml penicillin-streptomycin at 37°C. LA and ALA were dissolved in 0.1 N NaOH at a concentration of 20 mM. AA, EPA and DHA were dissolved in absolute ethyl alcohol at a concentration of 10 mg/ml. Stock solutions were filter-sterilized and diluted with cell culture media for use. The final concentrations of the solvents were 0.001 M of NaOH and 0.6% of ethyl alcohol that were found to have little effect on the growth of the cells.

### Cell viability assay

GES1, MGC and SGC cells were seeded in 96-well plates at a density of 10,000 cells per well and allowed to attach overnight, after which cells were supplemented with different concentrations of LA, ALA, AA, EPA and DHA. The doses of fatty acids tested ranged from 0 to 200 uM. 10 uM of antitumor drug methotrexate (MTX) was used as a positive control. After 48 h of incubation with fatty acids and methotrexate, medium was removed and treated with 20 ul MTT solution (5 mg/ml) at 37°C for 4 hours for assessing cell viability by measuring optical density at 492 nm after dissolving the dye in 150 ul of DMSO. The viability was defined as [OD (cells plate)-OD (medium plate)]/[OD (control cell plate)-OD (control medium plate).

### Flow cytometric analysis of apoptosis

The apoptotic rate of cells was detected using FCM with the Annexin V-FITC/PI double labeling method [27]. GES1, MGC and SGC cells in logarithmic growth phase were seeded in 6-well plates (Corning Costar) at a density of 100,000 cells/ml (3 ml per well). After adherence for 24 h, the medium was then replaced with refresh RPMI 1640 medium supplemented with different treatment reagents: without fatty acids (control), with 150 uM LA, 150 uM ALA, 180 uM AA, 180 uM EPA, 180 uM DHA and 10 uM antitumor drug MTX. After 48 h of incubation, the cells were harvested using trypsin, washed twice with cold PBS. The cell suspension (1 mL) was centrifuged at 2000×g for 10 min. After discarding the supernatant, the pellet was re-suspended gently in 400 uL Annexin V-FITC binding buffer and incubated with 5 uL Annexin V-FITC in dark at ambient temperature for 15 min. Subsequently, cells were incubated with 10 uL PI solution for 5 min on an ice bath in the dark. Cell apoptosis was subsequently performed by flow cytometry using Cytomics FC 500 MCL (Beckman Coulter, Inc.USA).

### Malondialdehyde (MDA) and superoxide dismutase (SOD) assay

The levels of MDA and the activity of SOD were determined by using commercial MDA and SOD assay reagent kits obtained from Nanjing Kaiji Bioengineering Institute (Nanjing, China) [28]. GES1, MGC and SGC cells supplemented with various fatty acids and methotrexate for 48 h were harvested using trypsin, and washed twice with cold PBS to remove excess trypsin. The cell suspension (1 mL) was centrifuged at 1000×g for 5 min. The supernatant was discarded; the pellet was re-suspended gently in 200ul PBS. After ultrasonication, the MDA content and SOD activity were determined and defined as corresponding value: Corresponding MDA (or SOD) concentrations (%) = C treatment / CCK×100%.

### Intracellular reactive oxygen species (ROS) generation assay

The generation of reactive oxygen species (ROS) was monitored using DCFH-DA method [16] with Reactive Oxygen Species Assay Kit (Beyontime Company, China). After freely passing through membrane into the cell, DCFH-DA without fluoresce itself, can be hydrolyzed by intracellular esterases to generate membrane-impermeable compound DCFH which is oxidized by intracellular ROS to fluorescent compound 2, 7-dichlorofluorescein (DCF). GES1, MGC and SGC cells that were supplemented with various fatty acids and methotrexate, after the treatment period, were centrifuged, cell pellet was collected and the same was re-suspended in serum-free PRMI-1640 medium containing 10uM DCFH-DA and incubated for 30 min in dark. The level of DCFH fluorescence was analyzed (excitation wavelength of 488 nm and emission wavelength of 525 nm) by SpectraMax M5, Molecular Devices.

### Determination of LXA4 levels

The LXA_4_ concentrations in the cell culture medium of gastric normal and cancer cells *in vitro* were measured by commercially available enzyme-linked immunosorbent assay (ELISA) kit [29] from BOSTER Company (Wuhan, China) according to the instructions of the manufacturer. The sensitivity by this assay was 1.0 pg/ml of LXA_4_. All measurements were done in duplicate.

### Fatty acid analysis of cells

GES1, MGC and SGC cells supplemented with various fatty acids and methotrexate, after the treatment period, were centrifuged and the cell pellet was collected and re-suspended in 500 μL distilled water and then 1 mL 5% hydrochloric acid-methanol mixed solution (v/v) to extract fatty acids. The collected solution was sealed immediately and then evaporated at 100°C for 3 h. After cooling to room temperature, 500 uL distilled water was added to each glass tube and extracted with 3 ml of hexane thrice by mechanical shaking [30]. The collected supernatant was evaporated to dry under N_2_ and the dried residue was dissolved in 100 μL of hexane for GC analysis.

For fatty acid analysis, the samples were injected into an Agilent 6980 GC system equipped with a DB23 capillary column (0.25 mm×60 m×0.25 μm), and a FID detector (Agilent Technologies, Palo Alto, CA, USA). Helium was used as the carrier gas with a constant flow rate of 1.0 mL/min. One μL of the sample was injected into the Agilent 6980 GC system. The column temperature was initially kept at 130°C for 1 min, and then elevated to 170°C at an increasing rate of 6.5°C per min, followed by 2.75°C per min to 260°C for10 min. Both of the interface and ion source temperature were 200°C.

### Oil red ‘O’ stain

To know whether the cells supplemented with various fatty acids form lipid droplets in the cytoplasm, the cells were fixed in 10% formalin after 48 hours of incubation with 180 μM of various fatty acids. Oil red ‘O’ solution that was prepared by dissolving 0.25 g of oil red O in 100 mL isopropyl alcohol by gentle heat at 56°C for 1 hour. The solution obtained at the end of 1 hour is allowed to cool and the cooled solution is filtered through a coarse filter paper that was used as the stock solution. The working solution was prepared by diluting 3 parts of the stock solution in 2 parts of double distilled water (stock solution: double distilled water = 3:2).

GES1, MGC and SGC cells were seeded in 24-well plates (Corning Costar) at a density of 10,0000 cells per well and allowed to attach overnight, then cells were treated in 1640 medium with 180 uM concentrations of LA, ALA, AA, EPA and DHA and 10 uM methotrexate (MTX). After 48 h incubation, the medium was removed and cells were washed three times with PBS and then fixed in 10% formalin for 10 minutes. The fixed cells were washed three times PBS and air dried for 20 minutes. These fixed cells were immersed in 1 ml Oil Red ‘O’ solution, and allowed to stand for 30 minutes. At the end of 30 minutes of treatment, cells were washed with distilled water three times and observed and photographed.

### Statistical analysis

Data obtained from the present study was expressed as mean ± SD, and were analyzed with SPSS 13.0 software. Significance of differences analyses between different groups were performed using a one-way ANOVA test.

## Results

### Effect of PUFAs on cell viability

**Figure 1:**
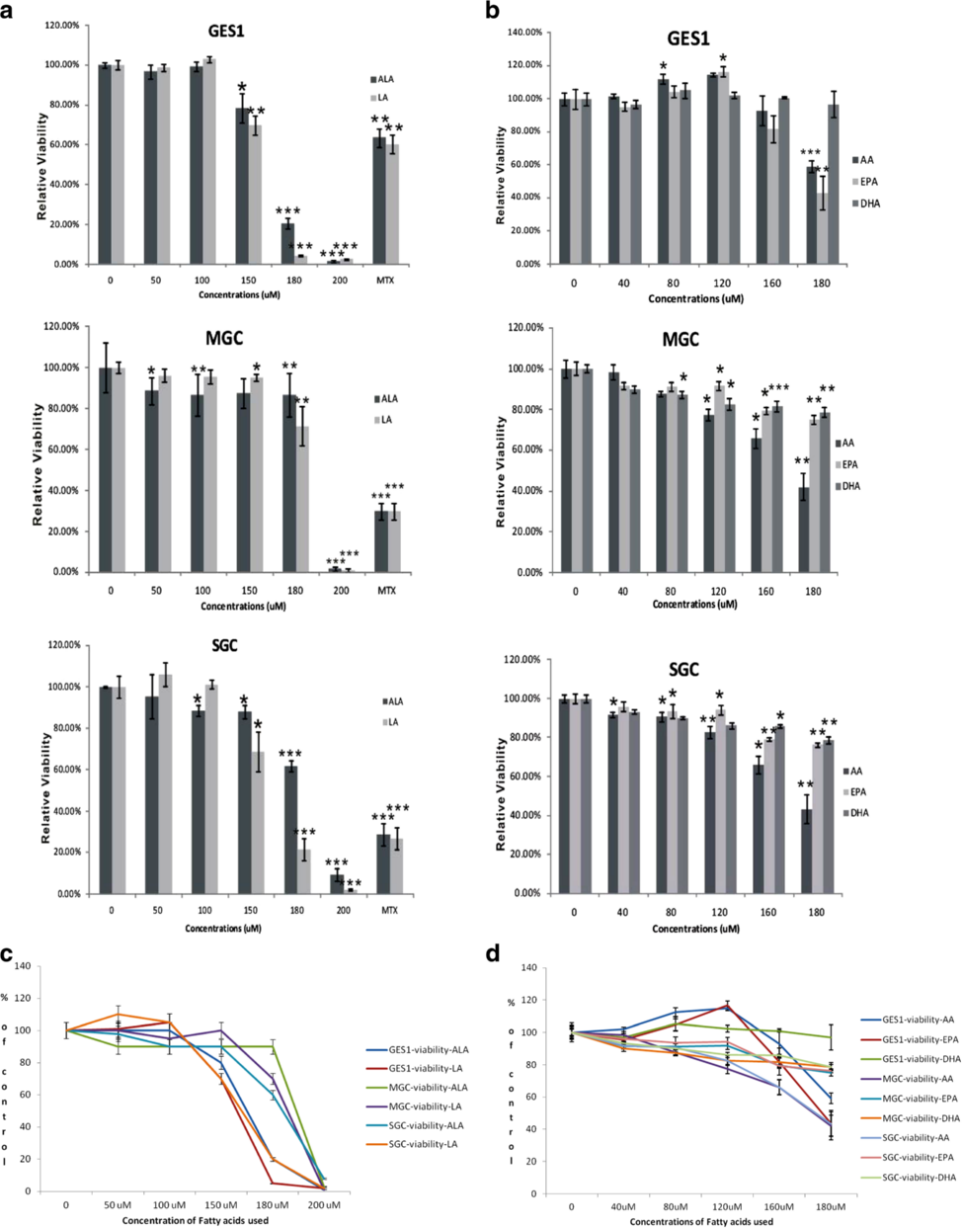
**Effect of various PUFAs on the survival of GES1, MGC and SGC cells in vitro. 1a. **Effect of LA and ALA on the survival of GES1, MGC and SGC cells. **1b.** Effect of AA, EPA and DHA and methotrexate on the survival of GES1, MGC and SGC cells in vitro. **1c. **Effect of ALA and LA on the survival of GES1, MGC and SGC cells *in vitro *on exposure to different concentrations of fatty acids. **1d. **Effect of various doses of AA, EPA and DHA on the survival of GES1, MGC and SGC cells in vitro. *P < 0.05; **P < 0.01; *** P< 001 compared to respective controls.

All the fatty acids (LA, ALA, AA, EPA and DHA) tested suppressed the growth and induced apoptosis of GES1, MGC and SGC cells at 180 μM and 200 uM. GES1 cells were found to be more susceptible to the growth suppressive actions of all the fatty acids tested except DHA compared with MGC and SGC cells. DHA, at a concentration of 180 uM, exerted little effect on GES1 cells while both MGC and SGC cells showed inhibited growth (see Figure 1). On the other hand, the common anti-cancer drug methotrexate (MTX) at 10 μM concentration showed significant growth inhibitory action on GES1, MGC and SGC cells (36.51%, 70.30%, and 71.04%, respectively).

### PUFAs induce apoptosis of cells

**Figure 2:**
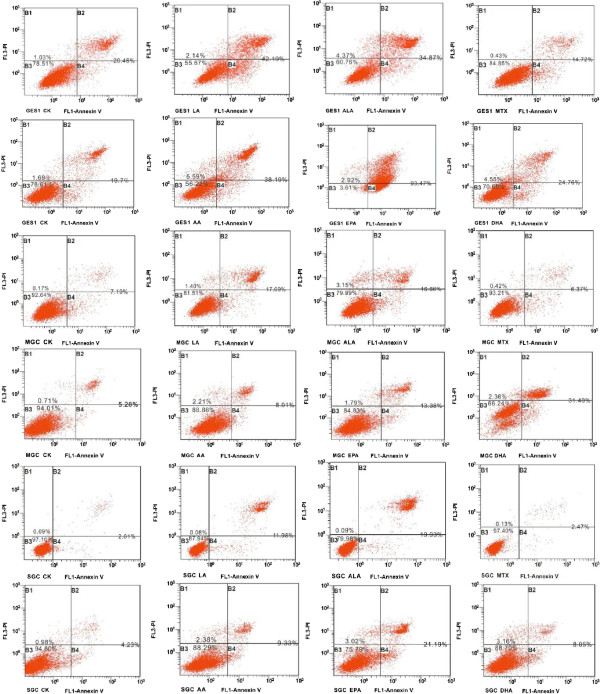
**Flow cytometric analysis of GES1, MGC and SGC cells treated with various PUFAs and methotrexate to determine their apoptosis on exposure to various treatments. **(150 uM LA and ALA on GES1 cell line, while AA, EPA and DHA were supplemented at 180 uM to GES1、MGC and SGC cell line; duration of incubation: 48 h.

The growth inhibitory action of PUFAs and methotrexate on GES1, MGC and SGC cells is due to their ability to induce apoptosis as determined by flow cytometry (see Figure 2).

### Effect of PUFAs on morphology of gastric normal and cancer cells

**Figure 3:**
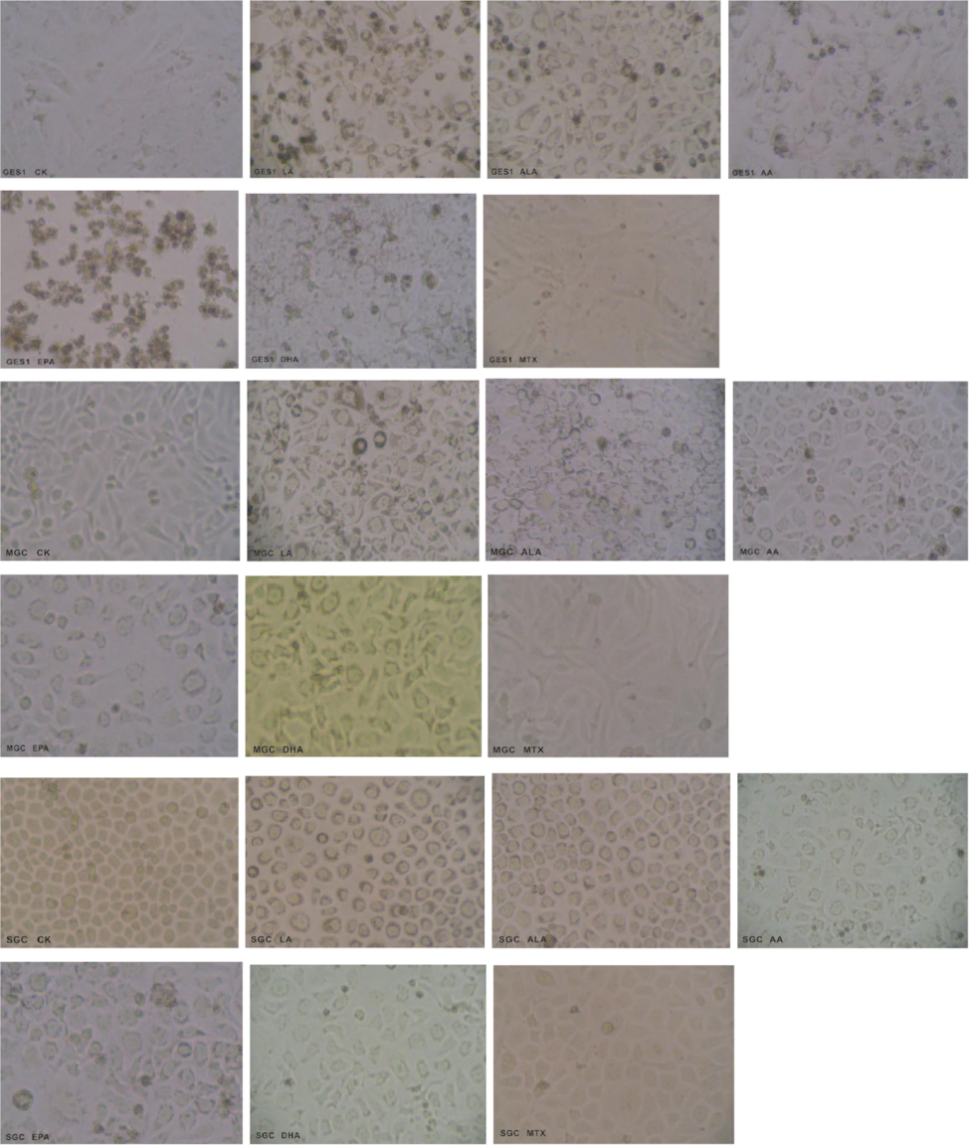
Morphological changes in GES1, MGC and SGC cells treated with various PUFAs and methotrexate (PUFAs: 180 uM; incubation time: 48 h).

Assessment of the cell morphology following exposure to various PUFAs showed accumulation of lipid droplets in the cytoplasm abutting the nucleus. As a result, the cells appeared to be larger in size and the densely arranged cells showed increased intercellular gap (see Figure 3). These changes in the cell morphology preceded apoptosis.

### Effect of PUFAs on oxidative stress

**Figure 4:**
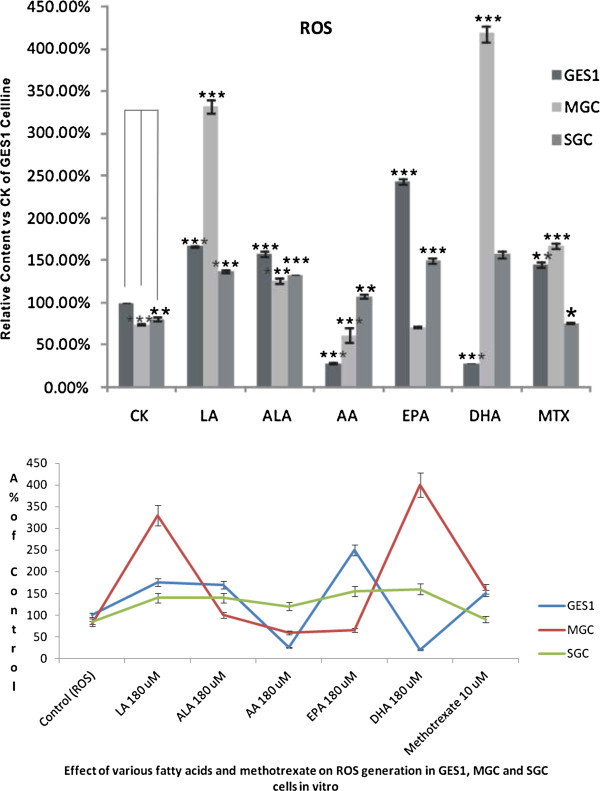
Effect of various PUFAs and methotrexate on the generation of ROS in GES1, MGC cells in vitro expressed as% of control. *P <0.05; **P < 0.01; ***P <0.001 compared to respective controls.

PUFAs are known to enhance oxidative stress and lipid peroxidation process. Under basal conditions, the amount of ROS generated by gastric normal cells (GES1) was much higher compared to gastric cancer (MGC and SGC) cells. When incubated with various PUFAs, the amount of ROS generated by GES cells showed significant increase except for AA and DHA (Figure 4). LA, ALA, and EPA, and MTX produced significant increase in ROS generation in GES1 cells compared to the control. In contrast, both AA and DHA decreased ROS generation in GES1 cells. These results are rather surprising since one would expect increased ROS generation in the presence of AA and DHA since they are highly unsaturated compared to LA and ALA.

On the other hand, ROS generation was significantly enhanced in MGC cells by LA, ALA and DHA and also by MTX. In contrast, AA and EPA did not produce any increase in ROS in MGC cells. In fact, both AA and EPA suppressed ROS production in MGC cells. In contrast, all the fatty acids tested produced a significant increase in ROS generation in SGC cells (Figure 4), whereas MTX reduced ROS production in these cells.

**Figure 5:**
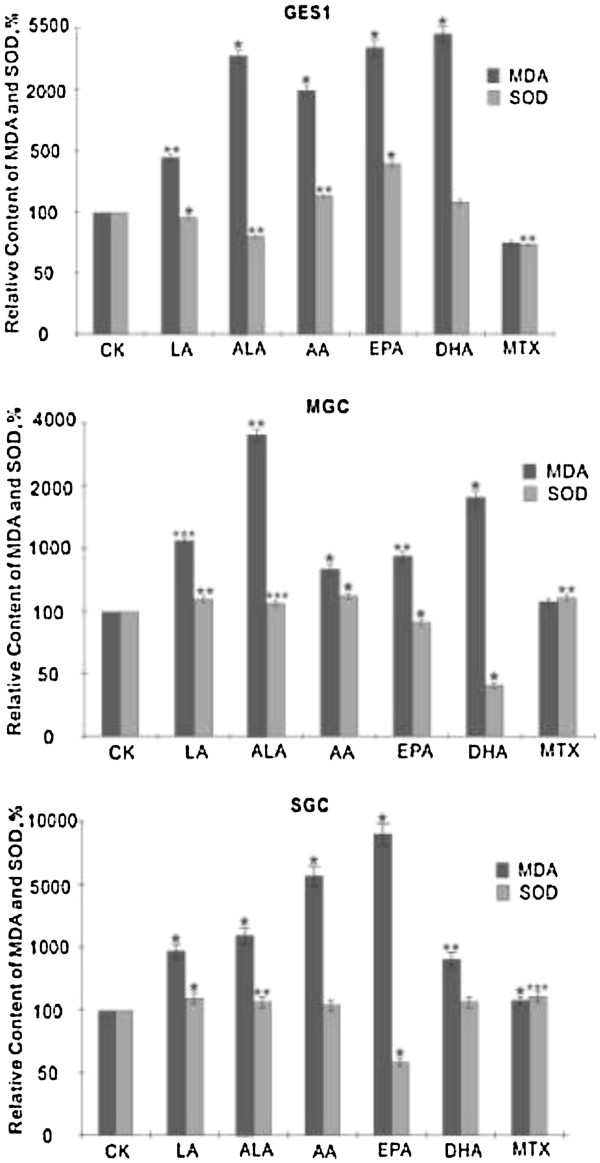
**Effect of various PUFAs and methotrexate on the concentration of lipid perpxides (measured as MDA) and SOD in GES1, MGC and SGC cells in vitro. **Cells were supplemented with 180 μM for 48 hours, while methotrexate was used at 10 μM for 48 hours. *P < 0.05; **P<0.01; ***P<001 compared to respective control.

The results shown in Figure 5 showed that MDA levels in both normal and cancer cells exposed to 180 uM of various PUFAs for 48 hours were significantly enhanced compared to the corresponding control group, while 10 uM MTX exerted much less effect on the MDA accumulation in all the three cell lines tested. In general, all PUFAs produced significant increase in the accumulation of lipid peroxides in the cells (in GES1 cells: DHA > EPA > ALA > AA > LA; in MGC cells: ALA > DHA > LA > EPA > AA; in SGC cells: EPA > AA > ALA > DHA = LA). These results suggest that the ability of various PUFAs to induce accumulation of lipid peroxides in different types of tumor cells could be substantially different. In contrast to this, the changes in the concentrations of SOD in the three cell lines tested to various PUFAs were quite different in comparison to the changes in lipid peroxides observed. Thus, GES1 cells showed highest increase in SOD in response to EPA though the highest increase in lipid peroxides was noted in response to DHA, whereas in MGC cells highest increase in SOD was noted in response to LA, ALA and AA while the highest increase in lipid peroxides was produced by ALA. In SGC cells, highest increase in SOD was seen on exposure to LA while highest increase in lipid peroxides was seen in response to EPA. Thus, increases in SOD activity in GES1 cells was as follows: EPA > AA > DHA > LA > ALA; in MGC cells: AA > LA = ALA > EPA > DHA; whereas in SGC cells: LA > DHA > ALA > AA > EPA (see Figure 5). This mismatch between the increases in lipid peroxides and SOD in PUFAs-treated cells suggests that the cellular response(s) to oxidative stress induced by various PUFAs are not uniform and are distinctly different that could reflect induction of apoptosis shown by different cells on exposure to PUFAs. In order to know whether the oxidative stress induced in cells exposed to various PUFAs depends on the ratio between the amount of lipid peroxides generated and the changes in the activity of SOD, we calculated ratio between mean values of lipid peroxides and SOD. These results shown in Table 1 revealed that ALA induced highest amount of oxidative stress in GES1 cells; DHA in MGC cells and EPA in SGC cells. When the relative amount of oxidative stress was calculated as LP/SOD ratio and correlated with the degree of apoptosis induced by 180 μM (the dose at which lipid peroxides and SOD were measured) of various PUFAs and methotrexate, it is clear that there was no direct correlation between the two (see Table 1).

**Table 1:** Calculated LP/SOD ratio and ROS generated in response to supplementation of 180 μM of various PUFAs and 10 μM of methotrexate for 48 hours by various cells

**Treat-ment**	**LP/SOD ratio in GES1**	**% of viable GES1 cells at 180 μM**	**LP/SOD ratio in MGC**	**% of viable MGC cells at 180 μM**	**LP/SOD ratio in SGC**	**% of viable SGC cells at 180 μM**	**LXA**_**4 **_**in the medium expressed in pg/ml GES1**	**LXA**_**4 **_**in the medium in pg/ml MGC**	**LXA**_**4 **_**in the medium in pg/ml SGC**	**ROS generated**		
										**GES% of C**	**MGC% of C**	**SGC% of C**
C	1.0	100	1.0	100	1.0	100	90	170	130	100	80	85
LA	5.0	4	7.4	70	3.9	20	125	125	100	175	325	140
ALA	50.0	20	27.3	90	13.1	60	135	120	150	150	150	150
AA	5.7	60	3.7	40	68.5	40	80	280	110	25	65	120
EPA	10.0	40	9.7	80	108.4	80	170	175	70	250	60	150
DHA	15.7	90	42.0	85	20.0	85	110	160	75	25	425	155
Metho- trexate 10 μM	1.0	60	0.63	30	0.91	30	120	110	100	150	160	70

C = Control.

### Effect of PUFAs on the generation of LXA_4_

**Figure 6:**
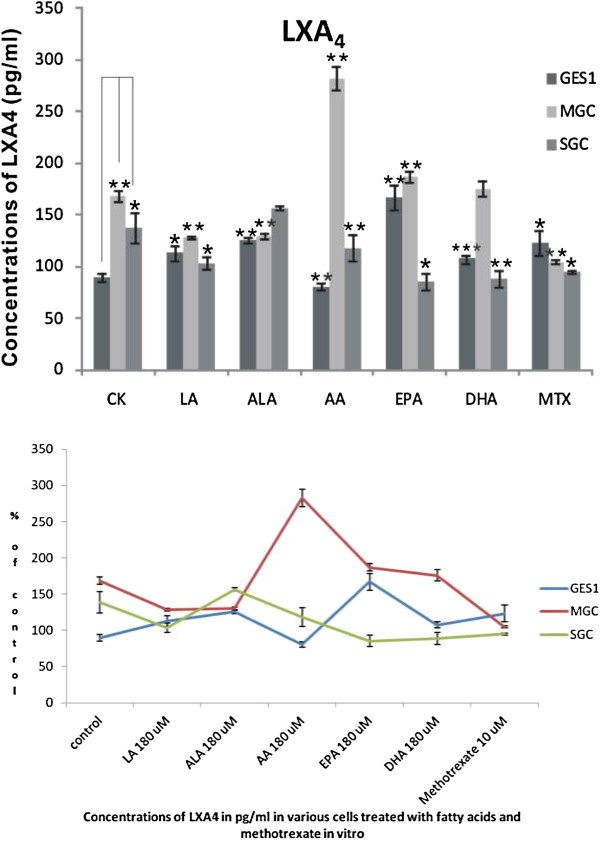
**Effect of various PUFAs (180 μM) and methotrexate (10 μM) on the content of LXA**_**4 **_**in GES1, MGC and SGC cells. **Time of incubation 48 hours. Effect of various fatty acids and methotrexate on the concentrations of LXA_4 _(in pg/ml) in GES1, MGC and SGC cells in vitro. *P <0.05; **P<001; ***P<0.001 compared to respective controls.

PUFAs not only form precursor to various PGs, TXs and LTs that are pro-inflammatory in nature, but also give rise to anti-inflammatory compounds such as LXs, resolvins and protectins. But, there are no reports that studied the effect of various PUFAs on the generation of these anti-inflammatory compounds by tumor cells. The results shown in Figure 6 clearly showed that all the three cell lines tested are capable of producing substantial amounts of LXA_4_ even under basal conditions. Supplementation of various PUFAs (180 μM for 48 hours) to GES1, MGC and SGC cells produced significant alterations in the synthesis and release of LXA_4_ in to the cell culture medium. Of all the fatty acids tested, supplementation of AA produced the most increase in the generation of LXA_4_ in MGC cells. Even under basal conditions, substantial differences in the amount of LXA_4_ generated by the three cell lines tested was observed (MGC > SGC > GES1). GES1 cells produced significantly enhanced amounts of LXA_4_ when supplemented with LA, ALA, EPA DHA and methotrexate and surprisingly produced much less LXA_4_ when supplemented with

AA (EPA > ALA > LA > MTX > DHA > Control > AA). The decrease in the production of LXA_4_ by GES1 cells in the presence of AA is rather surprising since AA forms the precursor of LXA_4_. In contrast, MGC cells produced significantly large amounts of LXA_4_ when supplemented with AA. Though other fatty acids were also effective in increasing LXA_4_ production in MGC cells, they were much less effective compared to AA (AA > EPA > Control > DHA > LA > ALA > MTX). Thus, in MGC cells only AA and EPA were able to enhance LXA_4_ production compared to control, while all other fatty acids decreased its synthesis. In SGC cells, ALA was the only fatty acid that enhanced LXA_4_ synthesis while all other fatty acids inhibited its production (ALA > Control > AA > LA = MTX > DHA > EPA). Thus, there was no consistent pattern with regard to the effect of various PUFAs on the production of LXA_4_ by GEs1, MGC and SGC cells. Nor we could find any correlation among formation of lipid peroxides, SOD, LXA_4_ formation and relative viability of cells when were supplemented with various PUFAs (see Table 1).

### Changes in the fatty acid composition of cells

It is known that both normal and tumor cells, when supplemented with PUFAs, incorporate the same in their phospholipid (PL) fraction. Hence, we analyzed PL fatty acid composition of GES1, MGC and SGC cells. Since the fatty acid analysis of the GES1, MGC and SGC cells was done twice (once while doing studies with LA/ALA and again while performing studies with AA/EPA/DHA), for uniformity, all the control values have been taken as 1.00 and accordingly the test values were readjusted so that comparison between the three groups of cells become easy and comparable. Results shown in Tables 2 and 3 revealed that there are significant alterations in the fatty acid composition of GES1, MGC and SGC cells when were supplemented with PUFAs. In general, the three cell lines showed substantial increase in the levels of respective fatty acids, especially of AA, EPA and DHA that were supplemented to the cells, though this is not the case always for all the fatty acids tested. For instance, GES1 cells when supplemented with LA and ALA did not show any increase in their LA and ALA content. On the other hand, when GES1 cells were supplemented with AA, EPA and DHA it led to substantial increase in their content of these fatty acids. It is noteworthy that GES1 cells supplemented with AA showed not only an increase in their AA content in the PL fraction but also a significant increase in LA, GLA, ALA, EPA and DHA (Table 2) with a concomitant decrease in palmitic, stearic and oleic acids. In contrast, both MGC and SGC cells when supplemented with AA did not show any significant increase in their AA content when fatty acid analysis of the PL fraction was done. This suggests that the uptake of AA by MGC and SGC cells is defective (or much less compared to GES1 cells) and/or the conversion of AA to their eicosanoid metabolites is significant so that AA content of the cells remained low. This needs to be verified in future studies by studying the metabolism of AA in these cells. In a similar fashion, supplementation of EPA and DHA to GES1 cells enhanced their PL content of these fatty acids by almost 4–8 times over the control while MGC and SGC cells did not show such an increase. Once again suggesting that, perhaps, MGC and SGC cells metabolize these fatty acids into their respective eicosanoids and other metabolites rather quickly. In table 3, a summary of changes in the fatty acid composition of GES1, MGC and SGC cells due to supplementation of various PUFAs is given for easy reference.

**Table 2:** Changes in the plasma PL fatty acids in GES1, MGC and SGC cells in response to supplementation of various PUFAs (180 μM) and methotrexate (10 μM) and after 48 hours of incubation

**GES1 cell line**	**CK**	**LA**	**ALA**	**AA**	**EPA**	**DHA**	**MTX**
Palmitic acid	1.00±0.00	1.28±0.04	1.15±0.02	0.72±0.03*	0.05±0.01**	0.78±0.05	0.94±0.08
Stearic acid	1.00±0.00	1.28±0.03 *	1.04±0.03	0.54±0.03*	0.03±0.01**	0.57±0.01**	0.96±0.10
Oleic acid	1.00±0.00	1.53±0.24	0.87±0.04	0.54±0.02*	0.69±0.01 *	0.67±0.01 *	1.76±0.07 *
Linoleic acid	1.00±0.00	0.90±0.13	0.86±0.02*	1.32±0.04*	0.19±0.02**	0.53±0.10	1.64±0.16
γ-linolenic acid	1.00±0.00	0.46±0.02 *	0.88±0.02	1.22±0.07*	9.52±0.25 *	0.22±0.07 *	0.70±0.10
Dihomo-GLA	1.00±0.00	0.94±0.08	0.92±0.04	1.07±0.39	0.31±0.01**	0.54±0.12	0.80±0.01 *
Arachidonic acid	1.00±0.00	1.01±0.13	0.90±0.07	3.50±0.08*	0.29±0.06 *	0.43±0.06	0.94±0.07
α-linolenic acid	1.00±0.00	0.85±0.01**	0.89±0.06	1.56±0.08*	0.88±0.11	0.48±0.23	0.88±0.01 *
EPA	1.00±0.00	0.93±0.03	1.00±0.05	1.27±0.01*	4.46±0.22 *	0.20±0.01***	0.84±0.06
DHA	1.00±0.00	1.36±0.08	1.59±0.07	1.72±0.03*	0.17±0.04 *	8.77±0.02 **	1.16±0.14
**MGC**	**CK**	**LA**	**ALA**	**AA**	**EPA**	**DHA**	**MTX**
Palmitic acid	1.00±0.00	0.89±0.01 *	0.84±0.04	1.64±0.18	1.42±0.09	1.82±0.20	0.44±0.03 *
Stearic acid	1.00±0.00	0.64±0.03 *	0.64±0.01 *	2.57±0.73^*^	1.71±0.06 *	3.13±0.78^*^	0.56±0.06
Oleic acid	1.00±0.00	0.36±0.01 *	0.35±0.01**	1.31±0.20	1.22±0.40	0.87±0.13	0.23±0.05 *
Linoleic acid	1.00±0.00	8.76±0.35 *	0.73±0.02 *	0.48±0.22	0.34±0.09	0.71±0.12	1.90±0.09 *
γ-linolenic acid	1.00±0.00	4.70±0.08**	0.89±0.19	0.50±0.02 *	3.15±0.39^*^	0.67±0.19	4.76±0.27 *
Dihomo-GLA	1.00±0.00	0.91±0.06	1.13±0.04	1.88±0.07 *	1.48±0.04 *	0.95±0.07	2.28±0.27 *
Arachidonic acid	1.00±0.00	0.42±0.01**	0.68±0.11	1.26±0.13	0.46±0.06 *	0.81±0.04	1.70±0.05 *
α-linolenic acid	1.00±0.00	0.53±0.11	8.62±0.65**	0.62±0.06	0.43±0.12	0.60±0.09	3.53±0.03**
EPA	1.00±0.00	0.83±0.01**	0.24±0.08 *	0.83±0.12	1.40±0.01**	0.78±0.05	2.63±0.03**
DHA	1.00±0.00	1.29±0.20	0.74±0.02 *	0.30±0.02*	0.31±0.09^*^	0.55±0.30	5.76±0.79 *
**SGC**	**CK**	**LA**	**ALA**	**AA**	**EPA**	**DHA**	**MTX**
Palmitic acid	1.00±0.00	1.00±0.04	0.92±0.02	0.95±0.07	1.32±0.19	0.45±0.06 *	0.91±0.12
Stearic acid	1.00±0.00	0.65±0.22	0.64±0.15	1.65±0.06 *	2.81±1.25	1.78±0.78	0.71±0.14 *
Oleic acid	1.00±0.00	0.75±0.11	0.71±0.14	0.61±0.24	0.18±0.01**	0.34±0.01 *	0.41±0.07
Linoleic acid	1.00±0.00	0.58±0.02 *	0.44±0.05 *	0.61±0.14	0.83±0.17	1.39±0.22	0.69±0.13
γ-linolenic acid	1.00±0.00	0.73±0.07	1.28±0.34	2.94±1.00	1.00±0.17	1.44±0.43	0.98±0.22
Dihomo-GLA	1.00±0.00	1.85±0.02**	2.41±0.29 *	2.22±0.35	1.04±0.08	1.77±0.19	2.11±0.25
Arachidonic acid	1.00±0.00	1.72±0.07 *	1.04±0.00	1.26±0.16	0.75±0.00***	1.50±0.23	1.32±0.16
α-linolenic acid	1.00±0.00	0.70±0.02 *	2.77±0.00***	1.35±0.72	1.45±0.14	1.55±0.45	1.41±0.15
EPA	1.00±0.00	2.86±0.03**	1.17±0.03	0.80±0.02	0.51±0.07^*^	1.11±0.06	1.53±0.13
DHA	1.00±0.00	1.24±0.15	0.50±0.01 *	0.28±0.01**	0.44±0.06 *	1.68±0.01**	2.18±0.34**

The basal value (control) was taken as 1.00.

*P < 0.05 compared to control (1.0). **P < 0.01 compared to the control; ***P < 0.001 compared to control.

**Table 3:** Plasma PL fatty acid profile of GES1, MGC and SGC cells supplemented with various fatty acids (180 μM) and methotrexate for 48 hours

**FA**	**Fatty Acid supplementation or Methotrexate supplementation**					
	**LA**	**ALA**	**AA**	**EPA**	**DHA**	**MTX**
	**GES1 MGC SGC**	**GES1 MGC SGC**	**GES1 MGC SGC**	**GES1 MGC SGC**	**GES1 MGC SGC**	**GES1 MGC SGC**
PA	↔ ↓ ↔	↔ ↔ ↔	↓ ↔ ↔	↓ ↔ ↔	↔ ↔ ↓	↔ ↓ ↔
SA	↑ ↓ ↔	↔ ↓ ↔	↓ ↑ ↑	↓ ↑ ↔	↓ ↑ ↔	↔ ↔ ↓
OA	↔ ↓ ↔	↔ ↓ ↔	↓ ↔ ↔	↓ ↔ ↓	↓ ↔ ↓	↑ ↓ ↔
LA	↔ ↑↑ ↓	↓ ↓ ↓	↑ ↔ ↔	↓ ↔ ↔	↔ ↔ ↔	↔ ↑ ↔
GLA	↓ ↑↑ ↔	↔ ↔ ↔	↑ ↓ ↔	↑↑ ↑ ↔	↓ ↔ ↔	↔ ↑↑ ↔
DGLA	↔ ↔ ↑	↔ ↔ ↑	↔ ↑ ↔	↓ ↑ ↔	↔ ↔ ↔	↓ ↑ ↔
AA	↔ ↓ ↑	↔ ↔ ↔	↑↑ ↔ ↔	↓ ↓ ↓	↔ ↔ ↔	↔ ↑ ↔
ALA	↓ ↔ ↓	↔ ↑↑ ↑	↑ ↔ ↔	↔ ↔ ↔	↔ ↔ ↔	↓ ↑ ↔
EPA	↔ ↓ ↑	↔ ↓ ↔	↑ ↔ ↔	↑↑ ↑ ↔	↓↓ ↔ ↔	↔ ↑ ↔
DHA	↔ ↔ ↔	↔ ↓ ↓	↑ ↓ ↓	↓ ↓ ↓	↑↑ ↔ ↑	↔ ↑↑ ↑

↔ = No change.

↑ = Increase.

↓ = Decrease.

↑↑ = Increase in the fatty acid concentration by more than 2–4 fold compared to control.

↓↓ = Decrease in the fatty acid concentration by more than 2–4 fold compared to control.

Supplementation of methotrexate to GES1, MGC and SGC cells produced few changes in the fatty acid composition of GES1 cells while MGC cells showed significant elevation in the PL content of LA, GLA, DGLA, AA, EPA and DHA, whereas similar increase in the content of these fatty acids was seen in SGC cells also but to a much less significant extent.

Tumor cells are known to have low activity of Δ^6^ and Δ^5^ desaturases [17-19]. Hence, we calculated the activity of these enzymes based on the levels of LA and AA and ALA and EPA seen in the three cell lines studied. The ratio between AA and LA (that is a reflection of the activities of Δ^6^ and Δ^5^ desaturases) was found to be 1.13 in GES1 cells, while the same was 0.05 in MGC cells and 2.97 in SGC cells (GES1 vs MGC vs SGC = 1.13 vs 0.05 vs 2.97 respectively). On the other hand, the ratio between EPA and ALA (that is a reflection of the activities of Δ^6^ and Δ^5^ desaturases) in GES1 cells was 1.13, in MGC cells 0.01 and in SGC cells 0.18. These results suggest that the activity of Δ^6^ and Δ^5^ desaturases is very low in MGC and SGC cells in comparison with GES1 cells (GES1 vs MGC vs SGC = 1.13 vs 0.01 vs 0.18 respectively).

### Formation of lipid droplets in fatty acid supplemented cells

**Figure 7:**
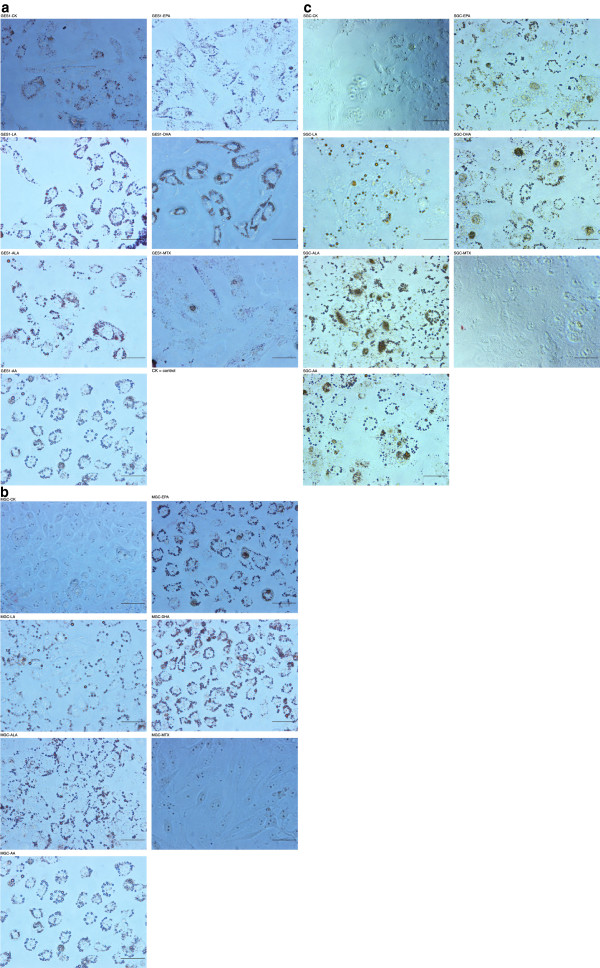
**Oil Red “O” staining of the lipid droplets that accumulated in cells supplemented with various fatty acids and methotrexate. **The cells were supplemented with 180 μM of fatty acid for 48 hours or 10 μM of methotrexate. CK = control. **7a**. Oil Red “O” staining of the lipid droplets that accumulated in GES1 cells supplemented with various fatty acids and methotrexate. The cells were supplemented with 180 μM of fatty acid for 48 hours or 10 μM of methotrexate. CK = control. **7b**. Oil Red “O” staining of the lipid droplets that accumulated in MGC cells supplemented with various fatty acids and methotrexate. The cells were supplemented with 180 μM of fatty acid for 48 hours or 10 μM of methotrexate. CK = control. **7c**. Oil Red "O" staining of the lipid droplets that accumulated in SGC clls supplemented with various fatty acids and methotrexate. The cells were supplemented with 180 uM of fatty acid for 48 hours or 10 uM of methotrexate. CK = Control.

Assessment of the cell morphology following exposure to various PUFAs showed accumulation of lipid droplets in the cytoplasm in these cells (Figure 7). Generally, cells supplemented with fatty acids accumulate them in triglyceride form in their cytoplasm that can be seen as lipid droplets. The accumulation of supplemented fatty acids as lipid droplets in GES1, MGC and SGC was confirmed by oil red “O” stain. It can be seen from these results that there is very little accumulation of lipid droplets in methotrexate treated cells while fatty acid treated cells showed significantly higher amounts and number of lipid droplets. Though it is difficult to quantitate the amount of lipid droplets that accumulated in GES1, SGC and MGC cells that were supplemented with various fatty acids, it can be seen from Figure 7 that, in general, ALA, AA, EPA and DHA supplemented cells showed more lipid droplets compared to the control and methotrexate treated cells.

## Discussion

Previously, we and others showed that PUFAs have cytotoxic action on tumor cells [3-16]. The mechanisms(s) by which PUFAs induce tumor cell death has been controversial. The suggested mechanism(s) of the tumoricidal action of PUFAs include: (a) increased generation of ROS; (b) enhanced lipid peroxidation resulting in accumulation of toxic lipid peroxide products in the cells that ultimately results in cell death; (c) activation of caspases; (d) activation of PPARs; (e) modulating gene/anti-oncogene expression, and (f) induction of chromosomal damage [1-16,31-37]. Though majority of evidences have been documented employing *in vitro* studies, some of these evidences have also been obtained using experimental animals [5-8,10,11]. Despite these evidences, it is still not clear as to the exact mechanism(s) of the tumoricidal action(s) of various PUFAs. For instance, majority of the studies employed cell culture techniques raising the question of their relevance to an *in vivo* situation. Furthermore, most of the *in vitro* studies were performed using only tumor cells without a simultaneous comparison or use of relevant normal cells. Thus, it is doubtful whether PUFAs are toxic only to tumor cells without being cytotoxic to relevant normal cells. This controversy could be answered by studying the effect of various PUFAs on the survival of normal and tumor cells employing similar, if not, identical cell culture conditions. In a previous study [1,2], we did show that some of the PUFAs especially γ-linolenic acid (GLA, 18:3 n-6), arachidonic acid (AA, 20: 4 n-6), eicosapentaneoic acid (EPA, 20:5 n-3) and docosahexaenoic acid (DHA, 22:6 n-3) does possess such selective tumoricidal action with little or no effect on normal cells. In these studies [1,2], it was noted that at higher concentrations (> 40 μg/ml/0.5 × 10^5^ cells) AA, EPA and DHA were toxic to normal cells (41-SK: human skin fibroblasts) in comparison to human breast cancer, prostate cancer and lung carcinoma cells. Of all the fatty acids tested, only GLA showed selective tumoricidal action. Despite this evidence, human skin fibroblasts cannot be taken as the normal counterpart of human breast cancer cells to conclude that GLA and other fatty acids are selectively toxic to tumor cells. In view of this uncertainty, in the present study we studied the effect of various PUFAs on normal gastric cells (GES1) and corresponding gastric carcinoma cells (MGC and SGC) and explored the possible mechanisms of their tumoricidal action.

It is evident from the results of the present study that all the PUFAs tested (LA, AA, ALA, EPA and DHA) and methotrexate were able to induce apoptosis of the three types of cells tested (both normal and gastric cancer cells) and showed very little differential action on normal and tumor cells (see Figure 1). Cell viability was affected only at higher concentrations (180 and 200 μM) of various PUFAs suggesting that both normal (GES1) and gastric tumor (MGC and SGC) cells are relatively resistant to the cytotoxic action of fatty acids tested. Such a relatively equal sensitivity of both normal and gastric tumor cells to the cytotoxic action of various PUFAs and methotrexate may explain why it is hard to treat gastric cancer since at doses at which anti-cancer drugs are able to kill tumor cells; perhaps, even normal gastric cells will also be affected. Such an equal sensitivity of both normal and tumor gastric cancer cells to the cytotoxic action of anti-cancer drugs could be the reason for various gastrointestinal side-effects observed during the treatment of gastric cancer. In the absence of a selective cytotoxic action of anti-cancer drugs on gastric cancer cells, normal gastric cells also bear the brunt of the actions of chemotherapy and lead to significant side-effects and complications in the management of gastric cancer.

One distinct observation that was made in the present study was the accumulation of lipid droplets in both normal GES1 and gastric tumor cells MGC and SGC that were supplemented with 180 μM of various fatty acids for 48 hours when stained with oil red “O” (see Figure 7 which shows lipid droplets in fatty acid supplemented GES1, MGC, and SGC cells and the corresponding control). In general, ALA, AA, EPA and DHA supplemented cells showed more number of lipid droplets compared to the control and methotrexate treated cells.

Studies into the mechanism(s) of cytotoxic action of PUFAs and methotrexate showed little correlation among cytotoxic action of PUFAs and methotrexate on GES1, MGC and SGC cells; production of ROS, formation of lipid peroxides, changes in the levels of SOD and LXA_4_ in these cells, suggesting that none of these mechanisms seem to be solely responsible for the cytotoxic action of fatty acids and methotrexate tested. Of all, only accumulation lipid peroxides seems to show the most correlation between the cytotoxic action of PUFAs and methotrexate on GES1, MGC and SGC cells and apoptosis. This indicates that formation of lipid peroxides in the normal and cancer cells on supplementation with various PUFAs and anti-cancer drug (methotrexate in the present instance) are the best predictors of their cytotoxic action. In the present study, we have not studied the effect of GLA and DGLA nor did we evaluate the affect of PUFAs on gene/oncogene expression. Such studies may give further insight into the mechanism(s) of cytotoxic action of PUFAs on normal and tumor cells. It is also important to evaluate the affect of combined action of PUFAs and various anti-cancer drugs on the survival of normal and tumor cells. Some of these studies are planned in the near future.

## Competing interest

Authors declare no conflict of interest.

## Authors’ contributions

WP, SS and UND conceived the idea, drafted the protocol and designed the experiments. JD and JS performed the studies. SS and UND interpreted the data. All participated in the drafting of the manuscript. All authors have read and approved the final version.
